# A qualitative study of dental internships in Saudi Arabia: moving beyond perceptions to the reality of the practices of dental interns

**DOI:** 10.1186/s12909-023-04802-3

**Published:** 2023-11-03

**Authors:** Mohammed Mahmoud Sarhan, Maram Ali M. Alwadi

**Affiliations:** 1 Department of Preventive Dental Sciences, College of Dentistry, Taibah University, 42353 Al- Madinah Al- Munawwarah, Saudi Arabia; 2 Department of Dental Health, College of Applied Medical Sciences, King Saud University, P.O. BOX 145111, 4545 Riyadh, Saudi Arabia

**Keywords:** Dental education, Medical education, Internship, Practices

## Abstract

**Background:**

Dental internships are a vital way for recent graduates of undergraduate dentistry courses to bridge the gap between study and clinical practice. Interns’ perceptions of dental internships have been explored in certain studies but the reality of the dental internship and dental interns’ practical performance has not been examined. Therefore, this study aims to explore the reality of the dental internship as a transitional stage after completion of an undergraduate course in dentistry.

**Methods:**

This qualitative research recruited 23 dental interns from Saudi Arabia’s Riyadh Province. To explore the reality of dental internships, the research relied on a performative knowledge approach to examine interns’ practices and performance. Diaries and semi-structured interviews conducted virtually were used to gather data across three months. The data was then subject to thematic analysis that applied an inductive strategy. The data analysis’s credibility and trustworthiness were verified using triangulation techniques, an audit trail and member-checking.

**Results:**

Five key themes concerning dental interns’ practices were identified in this research: exploration, addressing knowledge gaps, responsibilities, decision-making and social connections. The most significant findings reveal that dental internships go beyond clinical work to include certain personal and social aspects that dental interns undertake during their internships.

**Conclusion:**

The findings of this research indicate that more real-world, practical knowledge should be integrated into the curricula of undergraduate dentistry programmes. In sum, this work highlights the need for holistic dental education that encompasses not only the clinical development of interns and students but also other elements such as their personal and social growth. Moreover, this research reveals that a performative knowledge approach can help researchers to identify significant findings regarding the practical experiences of dental interns. This study has implications for dentistry and any other medical speciality education programme that involves an internship.

## Background

Dental education programmes differ significantly across the globe but they generally all involve clinical, practical and theoretical education [1]. The first year of a conventional undergraduate dental curriculum focuses on the development of knowledge [1–4]. This year emphasises didactic learning and is known as ‘preclinical instruction’ with ‘clinical instruction’ coming in the following years as more and more clinical training is delivered [1–4]. On graduation, new dentists must immediately confront a real-world workplace and the various challenges of dental practice [1–4]. Thus, managing the transition between supervised undergraduate study and independent clinical practice is vital for dental programmes worldwide [1–4]. As with all transitions, the transition experienced by new dentists involves a dynamic progression from a previous situation to a new one [5].

Given the above, most dental programmes worldwide require that students complete a rotatory internship as a means of transitioning from theoretical and practical (preclinical) study to clinical training [6, 7]. Dental programmes in different countries refer to these transitional periods in a variety of ways but they are all designed to achieve the same thing. UK dental graduates, for example, must complete ‘foundation training’, previously ‘vocational training’, which consists of a year of mentored training [6]. Foundation training occurs in general dental practices and is intended to give graduates the skills they need to independently practice dentistry [6]. To take another example, dental students in China spend their final year of dental school completing an ‘intern year’ where they receive comprehensive clinical training [7]. Similarly, in Saudi Arabia, to achieve full Dental Practitioner status, dental graduates are required to spend a year interning at different hospitals [8–11]. The primary purpose of Saudi Arabia’s dental internship programme is to allow graduates to apply and strengthen their clinical skills and knowledge and gain greater levels of responsibility in relation to delivering high-quality and safe dental care [8–10]. By completing this internship year, graduates are better able to move from undergraduate study, where their work is closely supervised, to independent clinical practice where they monitor their own professional practice [8]. Additionally, interns are encouraged to become involved in research throughout their internship year. Interns are motivated to develop and advocate dental innovations and refine their science-writing skills, which enhances their learning and nurtures their professional development [10, 11]. A ‘certificate of competence’ is provided to all interns in Saudi Arabia who complete their training year and satisfy a set of specific requirements [8–11].

A great deal of research has explored dental programme transition periods from a variety of perspectives [12–14]. In their qualitative study, Ali et al. [12] explored recent UK dental graduates’ transition to independent practice to evaluate the advantages and disadvantages of year-long mentored training in general dental practice. The study’s 16 participants, all different stakeholders in dental education, were interviewed about their views on various aspects of dental graduates’ preparedness for professional life such as the source of training gaps, the challenges of transitioning to professional life and suggestions for how graduates’ preparedness could be improved [12]. Further research conducted in the context of India examined the feelings, experiences and thoughts of dental interns, in particular, regarding their work in accident and emergency departments, with focus groups used to collect data [13]. This study found that Indian interns’ understanding of how to deal with medical emergencies was limited but they were eager to improve their skills and knowledge in this area [13]. The study thus suggested that undergraduate dental curriculums should pay greater attention to emergency dental management earlier in the programme [13]. Finally, a cross-sectional study by Ramalingam et al. [14] based on data gathered via questionnaires evaluated how dental interns perceived the specific clinical procedure of placing dental implants in patients who were medically compromised. They found that, excluding patients with hypertension and diabetes mellitus, interns believed that the placing of dental implants was incompatible with the majority of medical conditions [14].

The existing research emphasises the importance of understanding dental internships but exclusively from the dental interns’ perspectives and according to their perceptions of aspects of this transitional period, including its benefits, challenges, ability to prepare interns for clinical work and the clinical training they receive during the programme. However, little attention has been paid to the reality of dental internships as examined by what interns do and practice during this period, leaving aside their perceptions. Consequently, this research aims to explore the reality of the dental internship, i.e. what is being practised during the internship, as a transitional stage after completion of an undergraduate dentistry course. The findings of this research may reveal valuable insights into dental internships and may thus have implications for educators in dental and other medical fields whose training programmes feature internships. The use of qualitative research methodologies can deliver a more profound understanding of the study participants’ experiences.

## Methods

### Study design

This research applies qualitative research methods to both the data collection and data analysis processes. It forms part of a wider research project aimed at developing a comprehensive understanding of dental interns’ experiences and thus the reality of dental internships. This research aims to explore the reality of the dental internship as a transitional stage after completion of an undergraduate course in dentistry.

### Study context

This research was conducted in Saudi Arabia’s Riyadh Province. The study participants were recruited from dental interns with bachelor’s degrees in dentistry from two Saudi Arabian universities.

### Sampling strategy and recruiting

This research applied two sampling techniques, namely, purposive sampling and snowball sampling. Purposive sampling involved selecting dental interns who had completed a minimum of nine months of their year-long internship. Snowball sampling was then applied during the data collection phase. Here, the researchers asked the first participants to suggest other dental interns who may be interested in participating in the study. These individuals were approached in person at their place of work by the researchers to seek their participation. The researchers described the research to the participants and recorded their contact details to be used to coordinate interview times. Before the interview, the participants were sent a topic guide to reflect on and make some notes to remind them of any important points they wished to raise in the interview. As per Ritchie and Spencer [15], the data saturation point was reached after data was collected from 23 participants, and no additional participants thus needed to be recruited. The researchers determined saturation, concluding that no new themes or data were being provided in additional interviews. The 23-participant sample size was thus decided to be enough to deliver a thorough picture of the reality of dental internships.

### Study participants

In total, 23 dental interns participated in this research of which 15 were women and 8 were men. Three of the interns were 25 years of age and the remaining 20 were aged 24. They had all completed a minimum of nine months of their year-long internship. For data collection, 14 participants submitted diaries and 9 were interviewed.

### Data collection methods

Two data collection methods were used in this research, namely, diaries and semi-structured interviews conducted virtually. The preferences of the participants and which method they considered would allow them to give the most complete information determined which method was used. Virtual interviews rather than in-person interviews were the preference of all the participants who chose the interview option. The primary language of the interviews was Arabic but the participants had the freedom to also speak in English. The interviews lasted from 30 to 45 min. All the participants who chose the diary option asked to receive the diary entry prompts in English and to respond in English. It often took several days for participants to return their diaries. Data collection was conducted from March 2023 to June 2023 (three months).

### Data collection instruments and technologies

The virtual interviews were conducted via Microsoft Teams or Zoom as chosen by the participants. All of the participants who wanted to complete a diary chose to receive their diary prompts via WhatsApp, which was easy to access and could be frequently updated. The diary prompts were written in Microsoft Word and then sent via WhatsApp. For the interviews, the researchers drew on their academic experience to create a topic guide that could help the interviewees to freely discuss their experiences and optimise the interviews. Three open-ended questions were included in the guide to provide the interviewees with the scope to address any issues they felt were relevant to the research aim. The questions referred to the social, educational and clinical elements of the participants’ internships:

1. What are you doing during your internship on the social, educational and clinical levels?
2. Please recount some stories about the practical changes you have experienced during your internship (as opposed to your undergraduate studies) socially, educationally and as regards your clinical practice.
3. What social, educational and/or clinical practices are you engaged in during your internship that are new to you and not something you have done previously as an undergraduate?

During the virtual interviews, the interviewer asked the participants to elaborate on their responses when appropriate to provide further examples or detail. In subsequent interviews, the researchers used the insights they had gained in early interviews to refine the topic guide and add new questions. The interviews were all recorded using an audio recording device.

### Data processing

The interviews’ audio recordings were transcribed by the researchers and translated into English. The diaries were written in English and were thus ready to be analysed. The confidentiality of the participants was safeguarded using data management and security protocols. These protocols stopped unauthorised persons from accessing the data and anonymised the transcripts.

### Data analysis

The researchers agreed that thematic analysis would be conducted as per the steps recommended by Braun and Clarke [16–18]. According to these steps, researchers must first familiarize themselves with the data, then create initial codes, identify themes, review the themes, name the themes and write the research report.

The first author was primarily responsible for the data analysis, which began with the review of the first interview. Nonetheless, the research team decided to validate the data analysis through peer debriefing to guarantee its accuracy and credibility. Thus, following each step, the first author consulted the second author to substantiate the analytical process. To begin, the first author delved into the data that had been gathered, meticulously reviewing the interview transcripts and diaries to deepen his understanding of the data. Specifically, the author familiarised himself with the practices of dental interns and the general structures revealed by the data. When reviewing the data, the author noted key findings and his initial reflections on the patterns that were emerging. As he became more familiar with the data, the author began to create codes. The author then analysed each line of the transcripts, assigning the relevant codes to pertinent phrases. The codes were descriptive and were a means of classifying the data. Following the creation of the initial set of codes, the first author identified the themes in the data. That is, the coded sentences were reviewed to find any recurring ideas and patterns in the transcripts and diaries. This brought to light the potential themes in the dataset. After identifying possible themes, they were reviewed and refined by the first author to ensure that they were accurate representations of the data. The themes were continually compared with the primary data by the researchers to guarantee that they were solidly based on the practices of the dental interns. Once the review and refinement of the themes were complete, they were given meaningful, descriptive names by the researchers. These names were intended to represent the fundamental content of each theme to provide a clear, succinct picture of what it covered. When the themes and their corresponding names were confirmed, a thorough narrative was created by the researchers in the results section to provide a detailed explanation of the themes. This narrative was supported by the data, for instance, by quotes from the transcripts, to make the analysis more credible and provide examples of the themes. Computer software was not used to analyse the data in this qualitative research with the research team choosing instead to use a manual, paper-based method.

According to Braun and Clarke [16], researchers need to follow the steps of thematic analysis to refine the direction of their analysis. Specifically, these steps can be used to help the researchers to define what is considered a theme, whether theoretical or inductive thematic analysis should be used and whether constructionist or realist thematic analysis is relevant. The researchers must also decide whether the themes they are identifying are latent or semantic.

This research follows Braun and Clarke [16] in that the themes identified are considered to reflect the meaning of the data collected. Nonetheless, we also follow Braun and Clarke [16] and Nowell et al. [19] in that the frequency of the appearance of a theme in the data is not always considered an accurate reflection of its importance. The present research applies inductive thematic analysis where themes are drawn from the participants’ responses and not pre-prepared based on a review of existing educational or other theories. This method is in line with a realist thematic analysis approach as the researchers considered that the participants’ responses reflected their experiences and were not the product of cultural or social factors. Additionally, the themes identified were semantic as they reflected an analysis of the direct meanings of the responses of the study participants. The researchers’ analysis in this study did not explore anything beyond direct meaning, for example, the cultural and social factors that could impact the participants’ responses.

### Knowledge position

In this research, what constitutes knowledge is heavily influenced by ‘performative knowledge’ as described by Mol [20, 21]. This is a unique position on obtaining knowledge and understanding reality. Specifically, performative knowledge focuses on what individuals do rather than what they think or feel. It is their performance and not their perspective or perceptions that matter. Performative knowledge aims to understand reality by sidelining the psychological filters of study participants. Mol [20] argues that this approach can be used in qualitative studies by using interviews and/or observation to collect data. Observation allows researchers to watch and record participants’ actions in a natural environment, while interviews only involve asking participants to share stories about their actions and not about their thoughts or feelings [20]. This approach is used here to understand dental internships in terms of the interns’ actions rather than their perceptions. The interview questions reflect the performative knowledge approach as they focus on actions (i.e. what the interns are *doing* on the social, educational and clinical level in their internship) and ask the interviewees to share stories about the *practical* changes they have experienced. Also, during the data analysis, any feelings expressed by the participants (e.g. “I felt that I don’t need more clinical training”, “I felt that experience was important in the dental care field”) were excluded and not coded. Thus, this research considers knowledge to be reflected in the actions of the dental interns.

### Strategies to enhance trustworthiness and credibility

The credibility and trustworthiness of the data analysis were verified using triangulation, an audit trail and member-checking. To satisfy the triangulation technique, the researchers employed both diaries and interviews as data sources. Moreover, an audit trail was preserved to create a record of the researchers’ decision-making, data collection and data analysis processes. Finally, member-checking guaranteed that the researchers’ interpretations and the study’s findings were complete and accurate.

### Ethical considerations

The rights and privacy of the study’s participants were carefully considered throughout the research. The study was given ethical approval by the Institutional Review Board of Taibah University (TUCDREC/270223). The participants’ anonymity and confidentiality were safeguarded for the entirety of the research to keep their information private. Pseudo-anonymization procedures were used, such as assigning participants pseudonyms and changing any identifying traits to ensure confidentiality. Before the dental interns became participants in the study they received an information sheet about the study and were given a week to ask any questions they had. The research aim was repeated in the interview and the participants were again given a chance to ask any questions. The study’s participants provided written consent for their participation by signing a consent form after learning about the study. Where appropriate, O’Brien’s [22] guidance on reporting qualitative research is followed in this manuscript.

## Results

Several themes and sub-themes were generated by the analysis of the data gathered from the diaries and semi-structured interviews. The key themes identified were exploration, addressing knowledge gaps, responsibilities, decision-making and social connections.

### Exploration

The theme of exploration emphasizes two types of practical exploration available to interns during their internships: clinical exploration and personal exploration. These practices allow interns to explore a range of clinical settings and pursue personal interests, for example, travelling, developing new hobbies and starting a business.

### Clinical exploration

Internships allow interns to practice dentistry outside their university in various clinical settings to give them exposure to different knowledge, skills and experiences. Through the clinical exploration facilitated by internships, interns have diverse experiences in relation to patient care and have the freedom to explore different specialties and clinical environments. Interns visit different clinical settings where they can observe more experienced clinicians and learn from them about how to best care for patients. One participant (P4) discussed their experience of internship rotations in the following way:

*“ You’re right, the first four-month rotation was outside my university in one of the most common hospitals here. After that, I did a four-month rotation at my university hospital. We have around 20 or 19 clinical training centers in the internship that we can train at. After the four-month rotation at my university hospital, I was allowed to complete the rest of my internship there [at my university hospital], but I took another rotation outside the university. This was another different experience compared to the first and second ones. I joined them to see how they work there.”* (P4).

For this participant, their internship gave them repeated opportunities to practice dentistry at various facilities outside their university. A different participant (P6) spoke in more depth about one of their internship rotations:

*“First of all, I started in a hospital outside the university. It was my first rotation and someone recommended the periodontal treatment department there. I went there and got to know the people who were in charge and the residents and saw several operations related to periodontology, things I have not seen in my undergraduate degree, only discussed theoretically. I learned from them…I got to know them, I built a new family there. I got my clinical schedule from them and an aesthetic dentist was in one day and an endodontist in another day and a periodontist another day. I used to observe cases there.”* (P6).

According to this participant, their first rotation was completed outside their university. They spent several months at a clinical department focusing on periodontology but were also exposed to a variety of other specialties such as aesthetic and endodontic dentistry. The participant not only learned from their colleagues but also formed social connections with them.

### Personal exploration

Dental internships not only give interns the freedom for clinical exploration but also allow them to navigate and engage in personal exploration. During their internships, dental interns embark on new hobbies, travel, improve their physical fitness or even launch a business. Several participants (P21, P18, P17) discussed the personal exploration they have engaged in as interns:

*“I got to experience travelling with my friends during my internship.”* (P21).

*“I started a new business, I travelled, I got a personal trainer.”* (P18).

*“Talking about myself, during my internship, I started a new hobby, running. It started as a conversation between me and someone else at the gym who is a powerlifter. He is a coach at the gym. Basically, he gave me an application. I didn’t know what to expect from running…I started to run every day. I included it in my daily routine.”* (P17).

To conclude, internships give interns a valuable opportunity to engage in a process of clinical and personal exploration. In terms of clinical exploration, they can experience various clinical settings and watch and learn from oral healthcare clinicians who display a range of different approaches. Regarding personal exploration, internships give interns the freedom to pursue their interests and hobbies and find new ones.

### Addressing knowledge gaps

According to the data, internships are vital for helping dental interns to address the gaps in their education, especially as regards any advancements in knowledge that have occurred during their undergraduate dentistry course. Moreover, internships can help to fill the gaps in real-world, practical knowledge that is not covered in an undergraduate dentistry course. Dental graduates have been found to lack real-world, practical knowledge, but dental interns compensate for this lack through their clinical rotations where they work alongside experienced clinicians on real patients. As one participant (P1) explained:

*“Okay, in our undergraduate degree, we don’t have ‘clinical’ ortho, never, we only have lab and because of the coronavirus, even the lab exercises were done online. There was an ortho module in the undergraduate curriculum but not ortho in practice. The theoretical part was more than excellent, it’s enough, seriously, but the clinical side is missing…but when I went on rotations outside my university, I enjoyed them, I benefited from them. In those three months, I learned a lot. I saw cleft patients, surgical cases and functional devices. What was theoretical I saw in real life…I made up for the previous missing part in my ortho studies.”* (P1).

The undergraduate course that this participant is completing does not include clinical orthodontic modules, but the clinical training they were given during their internship made up for this gap in their education. They were able to observe key clinical elements (“cleft patients, surgical cases and functional devices”) and, in this way, gain the knowledge they were lacking. A further participant (P12) agreed that internships can help address knowledge gaps:

*“In my bachelor’s days, even if I studied gum treatment and even if I had clinical cases in my periodontic module, I was not fully aware of how gum disease patients are admitted to dental clinics and released from them in real life, what doctors do to the patient, offer them, how they deal with them, what kinds of pain that the patient can have with gum disease, how to properly diagnose the patient or different treatments. I learned new techniques [in my internship], some about implants. I learned completely new concepts like osseodensification that I did not learn about in my undergraduate studies, but I learned about them recently [from observations during my internship rotations]. The most important thing I learned [during my internship], truly, was how to manage the patient more than the procedure itself.”* (P12).

As with the previous participant, this participant states that certain clinical elements are not taught in their undergraduate dentistry degree, such as osseodensification and patient management; however, the internship provided them with this training and helped them gain and practice the necessary skills.

### Responsibilities

The theme of responsibilities concerns the responsibilities that dental interns embody and is divided into two sub-themes: clinical management responsibilities and patient care responsibilities.

#### Clinic management responsibilities

Practically speaking, what is required from dental interns is very different from what is required from undergraduate dental students. Dental students have limited responsibilities and can depend heavily on their instructors. They seek direction, guidance and help from their supervisors usually after each step of their practice. Dental interns, by contrast, have far more responsibility for their actions and work more independently. The additional responsibilities that dental interns have were commented on by one participant (P3):

*“Here, I’m number one, I manage the clinic, while as an undergraduate, everything relies on my clinical supervisors. I can’t do anything until I get the okay from my supervisors. During the internship, my supervisor is not a manager only a supporter when I need him. The patients are in my hands. In my undergraduate studies, my supervisors used to observe me, check on me while I worked on a patient every now and then, but now they don’t check on me. It is my clinic, it’s my patient.”* (P3).

This participant explains that, as an intern, they take on the role of a professional dentist where they do not have to check with their supervisors before acting. In contrast, as a student, they cannot take action without getting their supervisors’ permission. Also, their supervisors do not repeatedly check on their work as they do with students. This topic is addressed by another participant (P1):

*“We are truly being treated like doctors, there is no close supervision at all, the clinical supervisors do not roam beside us in our clinics, if I need her, I will consult her…As an undergraduate, I used to go outside to the waiting area and call the patient to my clinic, now the dental assistant checks my schedule and calls the patient for me. Before, I used to book an appointment for my patients, now, the receptionist does that for me. I am no longer a call centre.”* (P1).

This participant describes how different their clinical management responsibilities are now they are an intern. They explain that their clinical supervisors no longer closely supervise their work as they did when they were a student. Additionally, there are tasks that they are no longer responsible for. Specifically, as a dental intern, they are no longer responsible for calling patients into their office from the waiting area (this is done by dental assistants) or for booking appointments with their patients (this is done by the receptionists).

#### Patient care responsibilities

Alongside their clinic management responsibilities, dental interns embody patient care responsibilities in their practices. During the undergraduate study of dentistry, the focus is usually on course requirements. However, as interns, they make sure to integrate patient care into their work. In their clinical practice, they prioritize their patients’ preferences and comfort over their own needs and approach dentistry from a patient-centered perspective. The following interview response shows how one participant (P2) practiced patient care as a dental intern:

*“As an undergraduate, patients came to me for specific reasons, for restoration, for example. I would do it for them, so I did what they wanted and simultaneously completed the course requirements, but now, in the internship, there are no requirements, but there are other things. For example, there was an inpatient in the rehab centre who was unable to come to me. It took me too much time too to find her room, to find the bed number to reach her, and then I wanted to speak to her, to take a brief medical history and I found that she had had a stroke and was unable to speak. I was unable to understand what her complaint was…time passed and I had two options; either I helped her, and this meant staying beyond my working hours, or I report the patient’s situation and refer the case to the next clinician. I chose to stay and I sat with her. Her daughter was there. It took me one hour to take her medical history. She was barely able to talk. Like, I told you before, I am not a very patient person except I was then. I said to myself that I would stay to make sure the patient felt comfortable.”* (P2).

This participant took several steps to care for their patient and take on their patient care responsibilities. They chose to stay and work beyond their working hours to help their patient and she spent a great deal of time taking a medical history. A further participant (P13) shared a similar story regarding their patient care responsibilities:

*“A patient came to my clinic. It was not my best day. I was not feeling good that day, I was so tired, my mind was so busy, I had a lecture to present. You understand, I was in the clinic under pressure and the patient came to me with a restoration that was falling out…I was not required to treat him as this clinic was for emergencies only, so I could transfer him to another department, but I could do the procedure he needed for him…so, it was between being more comfortable myself or giving the patient a good result. I pushed myself. I told myself that I would do the procedure for him and see how things went. He liked my restoration and there was another one next to it. He told me he only wanted me to do it. He changed my mood completely.”* (P13).

Similar to the previous participant, this participant prioritized patient care. They could have referred the patient to another department but instead chose to care for the patient themselves and treat a restoration the patient had that was falling out.

### Decision-making

A core theme that emerged from the data was decision-making. Decision-making was central to the practice of dental interns. During their internships, dental interns gain exposure to various clinical settings and experiences and work with a variety of both dental professionals and patients. Internships prompt interns to reflect deeply on their career paths and the areas they wish to specialize in, prompting a period of profound self-reflection and, consequently, important decision-making. For some, an internship will help them discover their professional passion but for others, it could lead to a realization that their personal traits and ambitions do not match their professional goals. The need to make significant decisions about one’s professional future during an internship makes decision-making a key part of being an intern. The theme of decision-making is discussed by one participant (P5) in the following terms:

*“I did not know what speciality to proceed with. At the beginning of my internship, I was lost because I did not know what I wanted. Everyone knew what they wanted and were moving forward but I was still lost. I benefited from my observations during my rotations. I started to make a list of specialities that I was interested in and then, over time, I excluded them one by one until only one or two specialities remained.”* (P5).

The above participant’s actions displays significant decision-making behaviors. Unsure of which specialty to pursue, they first list all the specialties they are interested in and, over time, use their internship to observe the realities of working in these areas. They then start to remove specialties until they are left with only one or two to choose from. One participant (P11) goes into further detail about the decisions they took as an intern:

*“The event that made me change my direction as a clinician and prompted me to take another direction was when I did clinical observations during my rotations. I wanted to be a pedodontist but when I saw one pedodontist manage eight children per day, I decided it was too much effort and very tiring. It did not suit me at all. Then, I worked as a pedodontist. I made it through the first day, then the second day and then I spent three months in the role until I was sure that I no longer wanted to be a dentist. I truly don’t want to live this life [managing patients in clinics]. When I was an observer, I observed the pedodontist and she was in a prestigious role, it’s a great position, but when I was in the role for three months, I realised that this is not my life. I decided to change, to look at other more experienced colleagues in different fields.”* (P11).

This participant’s story again shows how decision-making is a key part of internships and is reflected in the practice of interns. This participant began their internship thinking they would specialize in pedodontics but the reality of this specialty prompted them to engage in intense decision-making regarding their career. They observed the reality of being a pedodontist and worked as one before deciding to work in a different field.

### Social connections

The social connections that interns establish during their internships emerged as a significant theme from the data and can be divided into two categories: professional connections and personal connections.

#### Professional connections

Internships are a time and platform for forging strong professional relationships. Internships provide interns with unique opportunities to work intimately with others from different professional levels and network with professionals. As a result, interns can forge valuable professional links with more experienced colleagues. One participant described this experience with a resident (P4):

*“One of the most important relationships I established was with a chief resident during my rotation. She was even more excited to teach me than I was to learn. She used to call me and say “Hey, come to my clinic on this day because we have a clinical procedure.” If I didn’t, she’d say “Hey, you should have come the other day, why didn’t you come?” She was very keen and was disappointed with me if I did not come when she suggested.”* (P4).

From this participant’s experiences, it is evident that dental interns make professional connections with more experienced colleagues during their internships. A further participant (P1) said something similar but added that internship rotations not only allow you to create professional connections with experts but also with fellow dental interns from different institutions:

*“I benefited a lot from the clinical rotations outside my university. I established relationships with doctors and several other dental interns from different universities. My four-month rotations outside my university were great experiences.”* (P1).

This participant benefitted from their internship by making professional connections with interns as well as doctors.

#### Personal connections

In addition to the professional connections discussed above, the qualitative data analysis reveals that dental internships also allow interns to pursue personal relationships. This is because an internship is a new stage in an intern’s life, one where they may seek out possible life partners. The sub-theme of personal connections was especially evident in one participant’s (P14) responses. This participant highlighted seeking personal connections as an important part of their internship experience:

*“During my internship, I started to look for marriage. This idea is always in my mind. Once I have the financial capability, I’ll start to look [for a wife]. Really, I already started this year. Yes, I already started to look for my life partner.”* (P14).

Another participant (P11) shared stories about their friends’ experiences finding life partners while they were completing their dental internships. This participant (P11) confirms that the actions taken by dental interns extend beyond the professional to include trying to find someone to settle down with:

*“I have some friends who got married during their dental internships. They are not my close friends but the idea of getting married during an internship does exist…Also, one of my friends told me that she went to her father, a leading consultant and doctor, and told him that if he met a nice boy he should tell her about him.”* (P11).

In sum, it appears from the data that dental interns use their internships to simultaneously seek out both professional and personal connections. Just as dental interns take steps to forge valuable professional connections, they also take action throughout their internships to find life partners.

## Discussion

The current work marks the first qualitative research to apply a performative knowledge approach to the exploration of dental internships with a focus on the practices of dental interns. Five key themes emerged from the data: exploration, addressing knowledge gaps, responsibilities, decision-making and social connections. Regarding the theme of exploration, the findings suggest that dental interns perform both clinical and personal exploration. Clinical exploration refers to the fact that dental interns practice outside their universities in diverse clinical settings where they learn from more experienced colleagues and are exposed to different skills and knowledge. In contrast, personal exploration refers to the fact that dental interns pursue and form new hobbies and interests during their internships. Dental interns also use their internships to bridge any gaps in their undergraduate education. They achieve this by observing others and engaging in activities that give them the practical experiences their undergraduate degrees did not. Moreover, the practices of dental interns reflect the new responsibilities they are given during their internships regarding clinic management and patient care. While supervisors are available if required, interns do not need to seek their guidance for day-to-day clinic management. Regarding patient care, the data reveals that interns prioritize patient care over their own needs. Furthermore, the research results reveal that dental internships represent a stage in dental interns’ education where they can make important decisions regarding their careers and the specialist areas they wish to work in.

Finally, dental internships are found to be a period during which interns forge professional connections with doctors and colleagues and seek out a romantic life partner.

Only a relatively small amount of the existing literature [12–14, 23, 24] has explored dental internships and dental interns’ experiences. Nonetheless, despite the limited number of studies, their aims and research approaches vary significantly. Shenoy KS et al.’s [13] qualitative study and Ramalingam et al.’s [14] quantativatve study, for example, aimed to understand dental interns’ experiences regarding specific clinical elements of their internships. The focus of Shenoy KS et al.’s [13] research was interns’ experiences and perceptions of emergency rotations while Ramalingam et al. [14] were interested in exploring the knowledge and opinions of interns regarding a specific procedure, namely, placing dental implants in patients who were medically compromised. A further qualitative study by Ali et al. [12] was more general in scope and sought to understand the benefits and challenges of internships for dental interns. The present work overlaps to some degree with Ali et al. [12] as it also applies a qualitative approach and is not exclusively concerned with the clinical elements of dental internships.

Importantly, the findings of the research in this area [12–14, 23, 24] address various elements relating to dental interns and their internships, ranging from clinical and professional to social and personal. To illustrate, Ramalingam et al. [14] are primarily concerned with clinical elements and how interns perform regarding a specific clinical procedure, while other research [12, 24] explores the personal and social elements as well as the clinical aspects of dental internships. For example, study [24] found that dental interns (74% of participants) consider the clinical experience they gain during their internships to be the most valuable part of this experience with social interactions (42% of participants) considered the second-most important and personal experiences considered one of the least valuable (13% of participants). However, as this was a quantitive study, it was not able to report any details regarding the personal, social and clinical experiences of the participants. In contrast, study [12] covers the personal, social and clinical aspects of dental internships. On the clinical side, for instance, it explored dental interns’ viewpoints regarding increased patient loads. On the social side, it [12] dicussed the establishment of professional relationships, and regarding the personal, it highlighted the importance of personal traits in the creation of personal relationships.

The findings of this research support the results of other studies [12, 24] that show that dental internships are not solely clinical experiences but also include strong and vital social and personal components. However, this study goes further to present the novel discovery of previously unacknowledged aspects of the personal and social components of internships. Specifically, the sub-theme of personal exploration reveals that dental internships are a period when interns explore new interests and hobbies outside the clinical space and pursue their personal interests. At the social level, this research also reveals that dental interns seek romantic life partners during their dental internships. This research was able to identify these findings because of the wide scope of qualitative methodologies and, in particular, the flexibility of the topic guide the researchers used to gather data, which allowed the participants the freedom to address a broad variety of their activities as interns.

According to the findings of this research, dental interns complete their internship programmes in various settings and centres where they can learn directly from seasoned healthcare professionals. Through these experiences, they acquire a variety of oral health skills and knowledge. This result is in line with the existing literature on dental internships [25, 26], which similarly reports that dental interns benefit from a valuable diversity of training contexts during their internships, as well as insights and guidance from experienced healthcare professionals.

The evidence reported in this research plainly shows that dental interns actively use their practical clinical knowledge in their internships. In this way, they can fill gaps in their knowledge that were not addressed in their undergraduate course. The existing literature [24, 27, 28] also finds that undergraduate dental students can have gaps in their knowledge of certain areas of oral health and dental practice. Importantly, the existing research [24, 27] finds that prosthodontics and minor oral surgery are insufficient in dental courses. Among vocational dental professionals, research [28–30] has also found that education and training in surgical extractions, endodontics, prosthodontics and orthodontics can be lacking. The present study confirms the need to improve the education and training of undergraduate dental students in key areas. The problem of persistently low knowledge and self-confidence among dental students in key areas has not been adequately dealt with in recent years [29, 30]. To address this, dental education institutions should consider the use of internships as the optimal way to allow their students to develop the necessary knowledge and skills. This will achieve the primary objective of internship or vocational training, namely, to enhance interns’ confidence in clinical practice [8, 9]. Interestingly, the vocational training students surveyed by Patel et al. [31] reported that their education had, in their opinion, sufficiently addressed the knowledge and skills that they needed. These opposing findings about the adequacy of the education and training dental interns receive before their internships may be due to variations in the undergraduate courses completed by the research subjects.

The importance of the role played by supervisors during dental internships and their availability to interns is recognised in this as well as the existing research [25]. However, the current study and existing work on this topic have explored different elements of internship supervision. For example, existing research [25] has found that dental interns’ learning experiences can be improved if the supervisors fully understand the learning outcomes of the internships. However, the dental interns surveyed in this research benefited from readily available supervisors but showed a reduced dependence on supervision. In particular, it was found that the interns assumed primary responsibility for clinical tasks related to the management of their dental clinics without actively seeking guidance from supervisors. This suggests that the subjects of this research may, to a large extent, have achieved their professional aims during their internship.

An important consideration when comparing this work to the existing literature is what was deemed to constitute ‘knowledge’ in the other studies, which impacted what type of data they collected. To a large extent, all the studies discussed [12–14, 23, 24] adopted the perception-based approach. The perception-based approach is very valuable and completely valid if it is used in the right way. However, it can be problematic if it is used inaccurately. Nonetheless, proper use of this approach can be found in the existing literature in study [12], in particular, in that participants’ perspectives on recruitment for internships were captured to reveal that heavy competition made recruitment challenging. This is an example of the valid use of the perception-based approach as one of the best ways to capture information on the challenges confronting participants is by asking them for their perspectives on the challenges they face.

Another example of how this approach is implemented in this area of study can be found in a study [13] on emergency dental rotations that found that, after completing the rotation, dental interns ‘felt’ sufficiently confident to manage emergency cases. Another example is seen in the discussion section of study [14] where it is stated that 78.1% of the dental interns surveyed ‘perceived’ that they had a good amount of knowledge regarding the placement of dental implants in medically compromised patients. It is worth noting that the findings of these two examples [13, 14] are interesting and informative. However, from a knowledge perspective, this approach is not ideal. The finding of study [13] that interns feel able to manage emergency cases following completion of an emergency internship rotation has some limitations. The obtained knowledge here could be correct but it could also be incorrect as it may not reflect the reality. In simple terms, yes, the interns ‘feel’ confident but a consultant may evaluate them in practice and find that there are, in reality, not competent to manage such cases. This limitation is the same for study [14], which argues that dental interns feel confident that they can place implants in medically compromised patients.

In contrast to the above, the approach applied here is based on performative knowledge. This approach avoids perceptions and feelings to a large extent if not completely. It directly captures the reality of what dental interns do in practice during their internships by asking them to recount real stories and conclusions are then made based on what the interns do in practice.

To better illustrate this approach in comparison with the perception-based approach, one can look at Ali et al.’s [12] study, which identifies establishing professional relationships as a theme. This study captures data on the participants’ perceptions to underline the importance of good communication and teamwork skills in establishing professional relationships. This result reveals the participants’ viewpoints and is thus valuable. However, it is suggested that the social connections theme identified in the present research using the performative knowledge approach has greater value. This is because this theme emphasises the actions taken by dental interns regarding their relationships and not their opinions of whether communication skills are vital for relationship-building. In particular, the social connections theme directly explores what interns do and finds that interns form connections with a variety of colleagues. In sum, the performative knowledge approach raises the quality of the knowledge obtained in this study as it is not concerned with the participations’ opinions on the value of communication skills to relationship-building (these may be right or wrong as they reflect a point of view) but instead goes directly to the point by capturing the action of dental interns establishing relationships with colleagues. The knowledge that interns establish professional connections is thus unquestionably correct. It is real; it is something that has happened and still happens.

All the findings of the current work reflect the performative knowledge approach. The theme of responsibilities is a particularly clear example. The data captured under this theme does not show whether the participants ‘feel’ that they have greater responsibilities as interns than they did as students, it shows the actions they take that reflect their new responsibilities. The research reveals that dental interns have a different role to play than they did as students regarding clinic management and patient care. Specifically, they do not need to seek guidance from their supervisors before taking action in their clinics and their supervisors do not monitor their work. Moreover, dental interns prioritize the care of their patients, sacrificing their own needs to make sure their patients are comfortable by, for example, working additional hours to do the work needed to help the patient or take a full medical history, as shown in the ‘ Results’ section. As regards the theme of decision-making, this theme again demonstrates how decision-making takes place as a practical process through the actions of dental interns. For example, one intern wrote a list of specialties to help them choose what career path suited them best, observing their colleagues and removing the specialties that did not appeal to them until they had only a couple left to choose from.

Other valuable and interesting studies [32, 33] in this area do exist but they are unlikely to add much to our understanding of dental interns and internships as they do not explore either the practices or perspectives of dental interns but assess internships from the perspective of ‘outsiders’. To illustrate, one study [32] examined the opinions of students on mandatory internship programmes. While such research can build on the existing literature in various ways, it offers limited insight into internships. Preferably, the need for compulsory internships should be evaluated by interns who have or are completing an internship. They experience internships first-hand and are thus the best people to ask about their value. Educational specialists may also be able to offer valuable insights into the advantages and challenges of internships but students who have no experience of mandatory internships are not in a position to provide useful data on this topic.

The present study and the existing literature on dental internships [12–14, 23, 24] cover many of the same research areas as the literature on medical internships [34, 35] and pharmacy internships [36, 37]. The clinical elements of internships is one area where these studies overlap. One study [35], for instance, examined the perceptions of medical interns regarding the clinical training they were receiving as interns and whether it was sufficient. The same topic has been explored concerning dental interns [14] who were asked their opinions on the adequacy of their clinical knowledge after their internship was complete. The difference between these two studies is the clinical procedures involved as one was concerned with medical specialties [35] and the other [14] with dental procedures. Relating to the same topic, research on pharmacy interns [37] concluded that internships helped interns to improve their professional and clinical skills. The confidence concept in internships has also been explored in relation to pharmacy interns [36] and dental interns [12]. As internships in the areas of pharmacy, medicine and dentistry have common objectives as well as similar training environments and approaches to supervision, studies on pharmaceutical, medical and dental internships will undoubtedly address similar topics. Consequently, knowledge-sharing and cross-disciplinary collaboration between researchers in these areas have the potential to enhance internship programmes and create better-trained professionals in various healthcare fields.

### Study implications

The findings of this research have several implications. This research finds that internships play an important role in bridging the gaps in dental interns’ knowledge and provide them with the education and training that was lacking from their undergraduate courses. Thus, the curricula of dental education courses should be reviewed to make sure that students are being taught real-world, practical knowledge. Moreover, this study’s results regarding the personal and social elements of dental internships stress the value of taking a holistic approach to dental education to ensure that social and personal as well as clinical development is addressed.

A further implication of the findings of this research concerns the value of developing a flexible topic guide to help capture data in qualitative research. This approach can help reveal unanticipated results as was the case in this research regarding the personal and social elements of dental internships. In addition to the above, this research validates the usefulness of the performative knowledge approach as a means of discovering integral findings about dental interns’ real-world experiences, for example, as seen in the findings on the themes of responsibilities and decision-making.

Finally, the participants of this research were dental interns in Saudi Arabia’s Riyadh Province currently completing a mandatory internship but the study findings have implications for a wider population. What happens in one sphere can inform what happens in another. This concept was discussed by Mol [20, 21] and Silverman [38] and their discussions on the notion of generalizability. To expand, where there is an internship programme, whether it be in medicine, dentistry or pharmacology, there is decision-making, responsibilities, training and so on. Minor variations between these contexts (dentistry, medicine, pharmacology) do not prevent the experiences and lessons learned from one context from being transferred to another, for example, from a dental intern deciding during their internship to specialize in orthodontics or periodontics to a medical intern choosing between family medicine and public health. Nor do these small variations prevent the transferability of the experiences of a dental intern in one country to a dental intern in another country. What matters here is the existence of, for example, the decision-making concept across all contexts. Conclusions can be drawn here and applied there, but the question is to what extent?

The results of this study and their current alignment with medical internship literature support this assertion. To illustrate, the theme of “Clinical Exploration” identified in this study discusses the opportunities that dental internships provide for interns to practice in various clinical settings beyond their universities, allowing them to learn new and varied knowledge and skills. The studies on medical internships [39, 40] reveal similar findings, namely that internships give interns practical experience and expose them to various duties (or rotations) in a range of contexts. Additionally, our results show that dental interns assume new responsibility for duties regarding clinic management and patient care. The medical internship literature [39, 41] reflects this finding, highlighting the broader span of responsibilities that medical interns adopt.

### Study limitations

It must be noted that there are two key limitations to this research. To begin, it does not capture data on the participants’ subjective experiences, information that could help to better understand the participants’ viewpoints regarding, for example, their needs and concerns. Also, the research only collected data using diaries and interviews. These collection methods are widely used and valid but observation could also have been used to enhance the study results’ validity. The study participants’ statements about their practices as interns (captured via diaries and interviews) could have been combined with the observation of their practices to strengthen the data that was collected.

### Suggestions for future research

The researchers of this study have one key recommendation regarding future research in this field. Increasing numbers of studies are being conducted on internships but the specific dimensions of internship programmes have still not been properly defined. Future research should thus aim first to clarify the dimensions of internship programmes, such as personal, social and clinical elements, and benefits and challenges, creating a valid theoretical framework that covers all aspects and dimensions of internships. Consequently, these dimensions could then be systematically explored through empirical research to enrich the literature in this area.

## Conclusion

This qualitative research adopts a performative knowledge approach, highlighting actions rather than perceptions, to explore dental internships and the practices of dental interns. Various themes were identified that reflect certain practical elements of dental interns’ experiences, namely, exploration, addressing knowledge gaps, responsibilities, decision-making and social connections. This research also identified specific personal and social activities that dental interns engaged in during their internships. These include pursuing interests and hobbies and looking for a romantic life partner. The paper then moves on to criticize certain studies’ methodologies and highlight some of the epistemological limitations of the existing literature, clarifying how this study overcomes these weaknesses. This research also stresses the value of internships in bridging the gaps in practical training in dental undergraduate education and the necessity for institutions and programmes to adopt a holistic approach to dental education so that courses of study address interns’ and students’ personal and social as well as clinical development. Importantly, the implications of this research go beyond dentistry and the research’s findings can be applied to internships in any medical specialty. Future research should attempt to clarify the dimensions of internship programmes to establish a valid theoretical framework so that subsequent studies can approach and explore internships based on a valid theoretical foundation. Overall, this is a study in medical education but also a lesson about knowledge. This work makes clear how valuable the performative knowledge approach can be in qualitative research as a means of discovering new, valid insights about dental interns’ practical experiences. These insights can then be used to help improve dental education institutions and courses.

## Data Availability

The datasets used and/or analysed during the current study are available from the corresponding author on reasonable request.
